# Prognostic Scores and Risk Stratification of Myeloproliferative Neoplasms: 2026 Updates

**DOI:** 10.3390/cancers18111725

**Published:** 2026-05-25

**Authors:** Noor Al-Zubaidi, Estela Ruiz, Natalia Curto-Garcia, Priya Sriskandarajah

**Affiliations:** Guy’s and St Thomas’ Hospital, London SE1 9RT, UK; noor.alzubaidi1@nhs.net (N.A.-Z.); estela.ruiz@carm.es (E.R.); natalia.curto-garcia@nhs.net (N.C.-G.)

**Keywords:** myeloproliferative neoplasms, risk stratification, prognosis, molecular

## Abstract

Myeloproliferative neoplasms (MPNs) are a heterogenous group of myeloid disorders including polycythemia vera (PV), essential thrombocythemia (ET) and myelofibrosis (MF). All these conditions can be associated with significant morbidity including increased risk of thrombotic events as well as reduced survival. Selecting therapy is dependent on patients’ risk assessment and thus prognostication is vital in the management of these conditions. There have been significant developments in prognostic and risk stratification models for MPNs over the last few years, including incorporation of molecular markers. This narrative review article aims to summarize these prognostic models and provide practical advice on how clinicians can utilize these tools to develop personalized treatment strategies for MPN patients.

## Simple Summary

Myeloproliferative neoplasms (MPNs) are a group of long-term blood diseases that affect the bone marrow. These include polycythemia vera, essential thrombocythemia, and myelofibrosis. Although these conditions differ, they can be associated with significant symptom burden, and in the long-term they result in an increased risk of disease progression and shortened life expectancy. Treatment decisions for MPN patients require careful consideration and thus accurate risk assessment is an important component of their management. Major advances have led to the development of multiple risk scores that predict outcomes to help patients and healthcare professionals decide on management approaches. However, it can be challenging to delineate which of these risk assessments should be used in clinical practice. In this narrative review, we aim to provide an overview of these risk prediction tools and a practical guide on how these can be utilized to support personalized treatment decisions for MPN patients.

## 1. Introduction

MPN disorders are caused by gene mutations affecting the JAK-STAT signaling pathway. These mutations lead to constant activation of the JAK2 pathway which controls hematopoietic cellular proliferation, survival and differentiation. The main driver mutations are *JAK2*V617F, *MPL* and *CALR*. The understanding of driver mutations has shifted MPN disorders into genetically defined cancers shaping diagnostic workup, prognostication and tailored treatments. Although MPNs share these common driver mutations, they differ greatly in symptom burden, predicted disease outcome and leukemic transformation risk.

Polycythemia vera (PV), essential thrombocythemia (ET), and myelofibrosis (MF) demonstrate distinct clinical, hematological, and histopathological characteristics [1,2,3,4,5]. PV is characterized by erythrocytosis, ET by sustained thrombocytosis, and MF by progressive bone marrow fibrosis, cytopenias, constitutional symptoms, and extramedullary hematopoiesis. These disease-specific features contribute to differences in symptom burden, thrombotic risk, leukemic transformation, and overall prognosis. 

Thrombosis is a leading cause of morbidity and mortality in the MPN population. The interplay between chronic inflammation and clonal hematopoiesis elevates the risk for both arterial and venous thrombosis. Notably, many patients present with a thrombotic event, with the risk remaining a lifelong concern for all patients [1,2,3]. 

Traditional risk scores for predicting outcomes are focused on clinical variables. With the move towards precision medicine and advances in identifying the mutational landscape of patients, newer risk scores have been developed to refine prediction of disease outcomes. It is important to accurately risk stratify MPN patients, as it can guide management and shift treatment goals based on this assessment. Importantly, risk stratification can help to mitigate complications associated with MPN, including thrombosis as well as disease transformation, which are associated with significant morbidity and mortality [2,3,4,5].

Risk stratification tools in MPNs can broadly be divided into thrombosis-risk models and survival/progression prognostic models [1,2,3,4,5]. Thrombosis-risk tools are primarily designed to estimate the likelihood of arterial or venous thrombotic events and guide decisions regarding antithrombotic therapy and cytoreductive treatment [1,2,3]. By contrast, survival/progression models aim to predict outcomes such as overall survival, leukemic transformation, and progression to myelofibrosis [4,5]. While some clinical and molecular variables overlap between these models, they address distinct clinical endpoints and therefore have different implications for patient management.

The increasing use of machine learning and artificial intelligence has also contributed to this exponential increase in the number of risk assessment scores for MPNs [6]. These typically include patient characteristics and disease phenotype as well as the presence of high-risk molecular markers. It can be challenging to navigate the different scores clinically. Furthermore, access to advanced molecular and genomic testing may not be readily available in certain healthcare settings. Therefore, the aim of this review is to help navigate the different scoring systems available for PV, ET and MF, and how these can be applied in daily clinical practice.

## 2. Polycythemia Vera

Polycythemia vera (PV) is a chronic myeloproliferative neoplasm characterized by clonal proliferation of the erythroid lineage, typically accompanied by leukocytosis and thrombocytosis, and occurring independently of erythropoietin stimulation [7]. In more than 95% of cases, PV is associated with the *JAK2*V617F mutation, and less frequently with mutations in *JAK2* exon 12, leading to constitutive activation of the JAK–STAT signaling pathway. The main complications of PV are arterial and venous thrombotic events, which represent the leading cause of morbidity and mortality [8]. Other complications include hemorrhagic events, microvascular symptoms, aquagenic pruritus, progression to post-PV myelofibrosis, and, to a lesser extent, transformation to acute myeloid leukemia [9,10,11].

### 2.1. Risk of Thrombosis and Bleeding

Thrombotic events are detrimental to the survival of PV patients. The classical stratification of thrombotic risk (Table 1) is based on age and a history of thrombotic events. The European LeukemiaNet defines patients younger than 60 years and without a previous history of thrombosis as low-risk, whereas all other patients are considered high-risk [1,11,12]. Conversely, other studies have shown increased risk for cardiovascular events for those above the age of 65 years [13].

There has been increasing interest in assessing the quantification of the *JAK2* mutant allele, as higher variant allele frequency (VAF) levels have been linked to increased risk of thrombosis in MPN, with an allele burden greater than 50% being associated with an increased risk of venous thrombosis. This was demonstrated in multicenter cohorts and meta-analyses, where the hazard ratio for venous events was markedly elevated in this group. Interestingly, this was less evident for arterial thrombosis, where classic cardiovascular risk factors (diabetes, dyslipidemia, history of arterial thrombosis) carried greater predictive weight than the *JAK2* allele burden [14]. The underlying pathophysiology may explain this difference: venous thrombosis in polycythemia vera (PV) is more closely related to blood hyperviscosity, leukocytosis, and endothelial dysfunction induced by the *JAK2* mutational burden. By contrast, arterial thrombosis is more dependent on atherothrombotic factors and cardiovascular comorbidities [2,5]. Therefore, the *JAK2* mutant allele burden is considered an independent marker for venous thrombosis, but not for arterial thrombosis [14,15,16].

Based on the above, in clinical practice, *JAK2* VAF could enable more precise risk stratification, particularly in patients considered low-risk according to conventional risk scores but who have a high mutational burden [15,17,18]. Furthermore, as well as risk stratification, the dynamic nature of *JAK2* VAF levels over the course of the disease can potentially be used to assess treatment response, as well as reflect disease progression. This was demonstrated in the MAJIC-PV study, which examined ruxolitinib versus best available therapy for PV patients with either resistance or intolerance to hydroxycarbamide. One of the major findings of this study was highlighting a link between molecular response and better clinical outcomes, with molecular response defined as a 50% reduction in *JAK2* VAF levels [19]. Therefore, *JAK2* VAF levels may influence therapeutic decisions, such as intensifying antithrombotic prophylaxis or initiating cytoreductive therapy. However, it is important to highlight that the current National Comprehensive Cancer Network and European LeukemiaNet guidelines do not currently recommend modifying treatment on the basis of the mutant allele burden, although future studies are planned to assess this further [20,21].

In addition to *JAK2*, mutations in the epigenetic regulators *DNMT3A*, *TET2* and *ASXL1* have been associated with an increased risk of cardiovascular disease, as well as thrombosis in MPN patients, due to their role in pro-inflammatory signaling [22,23]. Pasquer et al. (2024) further highlighted this through a recent study, where they proposed two novel prognostic systems, the Arterial Thrombotic Score (ARTS) and Venous Thrombotic Score (VETS) (Table 2 and Table 3) [24]. These models incorporated *DNMT3A* and *TET2* mutations as risk factors for arterial thrombosis, assigned significant weight to cardiovascular risk factors in the assessment of arterial thrombotic risk, and included a high *JAK2*V617F allele burden (variant allele frequency, VAF > 50%) as a risk factor for venous thrombosis [24].

The VETS demonstrated limited discrimination and thus may not outperform conventional risk stratification tools. Furthermore, these scores did not discriminate between the different MPN subtypes, which may affect their prognostic precision. However, the ARTS did emphasize the significant role of cardiovascular risk factors in managing PV patients. This was further highlighted in a study by Duminuco et al., who utilized the QRISK3 score to risk stratify PV and ET patients [25]. QRISK3 is a calculator used in the United Kingdom for the general population, which incorporates age as well as cardiovascular risk factors to estimate the 10-year risk of a cardiovascular event, with a score 7.5% or above being considered high-risk [25]. In this study, Duminuco et al. demonstrated that the QRISK3 score outperformed conventional risk tools in predicting thromboembolic events in both PV and ET patients, including those who would be otherwise deemed ‘low-risk’.

### 2.2. Overall Survival Prediction

Traditionally, survival in polycythemia vera has been primarily impacted by thrombotic events, which typically occur in the first few years after diagnosis. The highest rates of thrombosis occur shortly before or at the time of diagnosis, with arterial thrombosis occurring in 16% of patients and venous thrombosis in 7% of patients prior to or at diagnosis, decreasing over time with appropriate treatment [11]. Studies have demonstrated that thrombotic events can even precede the diagnosis by up to two years, underscoring the importance of early recognition and risk stratification [1,11].

However, as mentioned earlier, there is now increasing interest in the role of additional high-risk mutations on outcomes beyond traditional thrombotic risk factors. More than 50% of patients with polycythemia vera harbor non-*JAK2* mutations, and prognostically adverse mutations including *SRSF2*, *IDH2*, *ASXL1*, and *U2AF1*. These have been associated with worse survival and higher risk of progression to myelofibrosis or acute leukemia [10,26]. Rana et al. confirmed this in a study of the mutational landscape of PV. This group found that *SRSF2* mutation was associated with older age and leukocytosis, while *ASXL1* mutation was associated with younger age. Additionally, patients with PV with any additional adverse mutation had significantly shorter median overall survival of 8.8 years compared to 17.8 years for those without any adverse mutation [26]. This has since led to the development of mutation-enhanced prognostic scoring systems that integrate both clinical and genetic variables to better predict overall survival and disease progression [27]. One example is the Mutation-Enhanced International Prognostic Systems for PV (MIPSS-PV), a prognostic scoring system which incorporated *SRSF2* mutational status as well as age, leukocytosis and thrombosis history (Table 4) [27].

However, it is important to consider that not all centers will have easy access to molecular testing, and therefore prognostic scores have been developed which do not require this. These include both the International Working Group-Myeloproliferative Neoplasms Research and Treatment (IWG-MRT) as well as the triple A scores [11,28]. Both scores are summarized in Table 5 and Table 6 respectively.

It should be noted that the triple A (AAA) score (Table 6) was initially developed by Tefferi et al. as a prognostic tool to help stratify survival and risk in patients with essential thrombocythemia [28]. However, studies by Krecak et al. and Lekovic et al. were able to validate the prognostic impact of this score in PV patients, especially with predicting rates of time to thrombosis and overall survival in this cohort [29,30]. This is unsurprising given the increasing data identifying the Neutrophil-to-Lymphocyte (NLR) ratio as a potential prognostic marker in PV patients, with NLR ≥ 5 being associated with increased venous thrombotic risk and poorer outcomes [31,32].

### 2.3. Risk of Disease Progression

Leukemic transformation and progression to post-PV myelofibrosis are proven risks for PV. Tefferi et al. found the cumulative risk of leukemic transformation to be 2.3% at 10 years and 5.5% at 15 years, while another study by the Mayo Clinic demonstrated 3.9% risk at 20 years [33,34].

Grinfeld et al. developed an individualized prognostic model that integrated clinical, hematological and genetic variables with the aim of predicting overall survival and the risk of progression to post-PV myelofibrosis or acute myeloid leukemia [20]. Unlike conventional point-based prognostic scores, the Grinfeld et al. model uses a continuous multivariable approach that simultaneously incorporates clinical and genetic factors, allowing for personalized risk prediction without fixed categorical thresholds, as summarized in Table 7 [20].

We have summarized all risk scores discussed in this section in Table 8 alongside key variables as well as predicted outcomes.

## 3. Essential Thrombocythemia

Essential thrombocythemia is characterized by a persistently raised platelet count ≥ 450 × 10^9^/L in the absence of a reactive cause [35]. Patients with ET must have a thrombotic risk assessment as this guides management, with those stratified high-risk being offered cytoreductive therapy [21,35].

### 3.1. Thrombosis Risk

The classic risk assessment for predicting thrombotic risk in ET considers age, previous thrombotic history and platelet count to predict thrombotic risk. Furthermore, as discussed previously, cardiovascular risk factors can help delineate low- versus high-risk patients [35]. The International Prognostic Score in Essential Thrombocythemia-Thrombosis (IPSET-T) is the only risk calculator which incorporates cardiovascular risk factors, as well as age and prior thrombotic event, making it highly effective at stratifying ET patients [36]. A revised version of the IPSET (revised IPSET-T) incorporates the presence of *JAK2*V617F mutation, due to its strong association with increased thrombotic events in ET patients [37,38].

As discussed earlier, Pasquer et al. developed thrombosis scores (ARTS and VETS) which incorporated clinical and molecular variables to predict thrombotic risk in MPN patients, and highlighted the importance of cardiovascular risk factors as well as *JAK2* allele burden. Importantly, these scores were able to distinguish between arterial and venous pathophysiology in MPN patients, allowing for more personalized treatment strategies [24].

### 3.2. Overall Survival

Often considered to be the most indolent of MPNs, overall survival for essential thrombocythemia remains slightly lower than that for the normal population, with data from the Mayo Clinic reporting a median overall survival of 18 years while another study by Smith et al. found a more conservative figure of 12.1 years after median follow-up of 5.7 years [34,39].

Similar to PV, the presence of additional adverse mutations has been associated with inferior survival in ET patients, independent of conventional prognostic models [40]. The Molecular International Prognostic Scoring System for Essential Thrombocythemia, MIPSS ET, builds on this with another three-level risk assessment using four independent risk factors including age > 60, male sex, WBC > 11 × 10^9^/L and adverse risk mutations (*SF3B1*, *SRSF2*, *TP53*, and *U2AF1*) [27,41].

As mentioned earlier, the AAA score was developed using easily available blood count parameters to predict survival for ET patients. The AAA model (Table 6) demonstrated superior predictive performance compared to the original IPSET and was validated in an external cohort by Tosoni et al., with this model being able to predict overall survival as well as risk of thrombosis and major bleeding [28,42].

More recently, the triple A plus (AAA+) model was developed, which expands upon the original AAA score by incorporating additional host-related factors including absolute monocyte count, male sex, arterial hypertension, and history of arterial thrombosis [43]. The inclusion of cardiovascular risk factors in both triple A and triple A plus scores is unsurprising, as these are well known for their prognostic impact in terms of both overall and thrombosis-free survival in MPN patients [44,45]. The inclusion of monocyte count in the triple A plus score is based on previous data suggesting a role of monocytes in driving inflammation in MPN, which has translated clinically with poorer outcomes and reduced survival reported in PV and primary MF (PMF) patients [46,47,48]. This was recently validated by Sönmez et al. (2025), who found elevated NLR as well as monocytosis (>0.8 × 10^9^/L) were associated with higher risk of mortality, and incorporation of the latter in the triple A plus score enhanced its prognostic accuracy [49]. Overall, these studies highlight that both the AAA and AAA+ models can be used effectively to risk stratify patients, relying solely on complete blood count-derived parameters and clinical factors, making them globally applicable and practical for initial assessment in settings where molecular testing may not be readily available [28,43].

### 3.3. Disease Progression

The risk of ET progressing into acute leukemia is generally low with increasing risk over time. In a study by Barbui et al., leukemia transformation rates were 0.7% at 10 years and 2.1% at 15 years while the risk of progression to overt myelofibrosis was 0.8% at 10 years and 9.3% at 15 years [50]. Multivariable analysis of this study did identify age ≥ 60 years, prefibrotic morphology, leukocyte count > 10 × 10^9^/L and thrombosis as independent risk factors for overall survival, while risk factors for leukemia-free survival included prefibrotic morphology, thrombosis and extreme thrombocytosis (>1000 × 10^9^/L). Notably, presence of *JAK2*V617F mutation was associated with lower risk of transformation to myelofibrosis [50]. A subsequent study which primarily focused on World Health Organization (WHO)-defined ET identified age ≥ 60 years, history of thrombosis and leukocyte count > 11 × 10^9^/L as risk factors for survival, underscoring the importance of accurate histological diagnosis of this condition [51]. Recently, a Mayo–Florence study identified triple-negative driver mutation status to be an overall favorable risk factor for ET in terms of fibrotic progression, arterial/venous thrombosis risk and risk of leukemia transformation. By contrast, both *MPL* and *CALR-1* were associated with increased risk of fibrotic progression, while *JAK2* was associated with increased risk of both arterial and venous thrombosis [52,53].

We have summarized all risk scores discussed in this section in Table 9 alongside key variables as well as predicted outcomes.

## 4. Myelofibrosis

Myelofibrosis is characterized by clonal proliferation of atypical megakaryocytes and granulocytes, leading to progressive bone marrow fibrosis, ineffective hematopoiesis, and extramedullary hematopoiesis, predominantly affecting the spleen and liver. MF may arise de novo as primary myelofibrosis or evolve from antecedent polycythemia vera or essential thrombocythemia [35]. The disease is driven in most cases by mutations in *JAK2*, *CALR*, or *MPL*, resulting in constitutive activation of the JAK–STAT signaling pathway, with additional somatic mutations contributing to disease progression and clinical heterogeneity [54,55]. 

MF is associated with a highly variable clinical course, ranging from indolent disease to rapid progression with severe cytopenias, symptomatic splenomegaly, constitutional symptoms, thrombotic and hemorrhagic complications, and an increased risk of transformation to acute myeloid leukemia [55]. Given this marked heterogeneity, accurate prognostic stratification is essential to guide therapeutic decisions, including the use of JAK inhibitors and the timing of allogeneic hematopoietic stem cell transplantation (allo-HSCT) [56]. To this end, several prognostic scoring systems incorporating clinical, hematologic, cytogenetic, and molecular variables have been developed [57].

### 4.1. Thrombosis Risk

Although the incidence of thrombosis is lower than in PV, both arterial and venous thrombotic events significantly contribute to morbidity, with one study reporting incidence of 13.2% at or prior to diagnosis, and 10.7% over median follow-up of 31 months [58]. In another study, fatal and non-fatal thromboses were diagnosed in 7.2% patients, although the overall death rate due to cardiovascular events was low at 2% [59]. This is likely due to other fatal non-cardiovascular events occurring in MF patients, including transformation to acute leukemia [60].

Venous thrombotic events commonly occur in unusual sites in MF patients, with one series by Cervantes et al. reporting 20% of MF patients with splanchnic vein thrombosis (SVT), while the German SAL-MPN registry reported SVT in 18% of MF patients [61,62]. Overall, these studies highlight that although incidence rates are low, thrombotic events remain a clinically relevant issue in MF and should be managed accordingly. Thus, risk scores have been developed to include MF patients, with the ARTS and VETS scores, as previously discussed, being a good example (Table 3 and Table 4) [24].

### 4.2. Overall Survival

The International Prognostic Scoring System (IPSS) and Dynamic International Prognostic Scoring System (DIPSS) are classical prognostic scoring systems in myelofibrosis, mainly used to estimate overall survival (Table 10) [63,64]. The IPSS is applied at diagnosis, whereas the DIPSS can be used at any time during the disease course; its main distinction is that anemia carries greater prognostic weight in the DIPSS [64]. This is unsurprising as previous studies have demonstrated worse outcomes as well as increased risk of disease progression in MF patients with increasing anemia severity [65,66]. The DIPSS-Plus model further refined risk stratification by incorporating cytogenetic data, along with additional clinical variables, in particular transfusion dependence (Table 10) [67]. The latter has been associated with an eightfold increased risk of death in MF patients and was a strong predictor of survival in multivariable analysis irrespective of the IPSS and DIPSS score [68,69]. Thrombocytopenia is also included in the DIPSS-Plus score as this has been associated with poorer survival outcomes, with severe thrombocytopenia (<50 × 10^9^/L) being particularly challenging due to limited treatment options [70,71].

Recently, Tefferi et al. developed the revised DIPSS score (DIPSS-R) excluding age and constitutional symptoms from the variables, utilizing disease-related factors more than host-related factors. Importantly, the aim of this score was to demonstrate that chronological age may not reflect biologic age, with DIPSS-R showing a similar 10-year predictive risk compared to the DIPSS score [72].

Allogeneic hematopoietic stem cell transplantation is the only potentially curative treatment for myelofibrosis but carries significant transplant-related mortality (TRM), with 1-year non-relapse mortality rates of approximately 15–26% and 5-year rates of 21–37% [73]. This substantial risk necessitates careful patient selection and accurate prognostic stratification to identify those most likely to benefit from transplantation while minimizing treatment-related complications [56,57,73].

More recent models, the Mutation-Enhanced International Prognostic Score System for transplantation eligible-aged patients with overt PMF (MIPSS70) and MIPSS70+ version 2.0, were specifically developed for patients aged ≤70 years and integrate molecular information, including high-molecular-risk (HMR) mutations (*ASXL1*, *EZH2*, *SRSF2*, *IDH1/2*, and *U2AF1 Q157*), with clinical factors [74,75]. However, MIPSS70 and MIPSS70+ version 2.0 have important limitations that must be considered when making transplant decisions. While these models include *ASXL1*, *EZH2*, *SRSF2*, *IDH1/2*, and *U2AF1 Q157* as HMR mutations, they do not incorporate other high-risk mutations that have been shown to significantly impact outcomes, particularly in the transplant setting, for example *TP53* mutation. Gagelmann et al. highlighted the impact of *TP53*, with their study demonstrating poorer outcomes with multi-hit compared to single-hit and wild-type, with 6-year survival rates of 25% vs. 56% vs. 64% respectively [76]. However, regardless of this, MIPSS70+ version 2.0 is the most comprehensive model available, combining clinical, cytogenetic, and molecular data, and therefore can help support therapeutic decision-making, including consideration for allogeneic stem cell transplantation (Table 11).

The Myelofibrosis Secondary to PV and ET-Prognostic Model (MYSEC-PM) is a prognostic model specifically developed for patients with secondary myelofibrosis arising from polycythemia vera (PV) or essential thrombocythemia (ET). Unlike classical scoring systems designed for primary myelofibrosis, MYSEC-PM provides a more accurate estimation of overall survival in this specific patient population by integrating readily available clinical variables [77]. Importantly, this score has been validated in secondary myelofibrosis patients treated with ruxolitinib, and maintained prognostic accuracy compared to IPSS in this same cohort [78]. Recently, MYSEC-PM has been refined further with the incorporation of specific genomic and cytogenic profiling (known as MYSEC-kmPM). This improved survival prediction in secondary MF patients, and outperformed MYSEC-PM [79].

The RR6 (Response to Ruxolitinib after 6 Months) score was developed in the RUXOREL-MF study to predict survival in myelofibrosis patients after 6 months of treatment with ruxolitinib [80]. This score incorporated dose of ruxolitinib, reduction in spleen size and red blood cell transfusion requirements after 6 months of treatment to risk stratify patients into three categories (low, intermediate and high risk). The key aspect of using this score was to allow for early identification of MF patients on ruxolitinib treatment who may benefit from an early treatment change in order to improve outcomes.

Machine learning has helped create several prognostic scores. The Spanish MPN group used machine learning and artificial intelligence to develop the Artificial Intelligence Prognostic Scoring System for Myelofibrosis, AIPSS-MF [81]. This score was developed to predict overall survival and leukemia-free survival for primary and secondary myelofibrosis. Based on eight clinical variables at the point of diagnosis, the model showed good prediction of overall survival and leukemia-free survival. These variables included age, sex, hemoglobin, leukocytes, platelets, peripheral blasts, constitutional symptoms, and leukoerythroblastosis. The key advantages of this model were that it provided personalized risk assessment for MF patients, and its predictive accuracy surpassed the IPSS and MYSEC-PM. Importantly, the model was based on clinical rather than genomic data, meaning it could be used more widely in clinical settings [81].

The same group subsequently looked at studying the impact of adding molecular profiles to their prognostic score (AIPSSmol-MF). Overall, they found that incorporating molecular data was associated with modest improvements in predicting overall and leukemia-free survival in MF, with the following genes having the greatest impact: *TP53*, *EZH2*, *IDH1*, *RUNX1*, *CBL*, *SRSF2* and *IDH2* [82]. This model outperformed the IPSS and MIPSS70 in predicting overall survival, and overall represented a more accurate and individualized approach to managing MF patients [82].

### 4.3. Disease Progression

Prognosis and treatment goals change significantly when there is progression to acute leukemia; hence, predicting this risk is critical. The MPN Personalized Risk Calculator, by Grinfield et al., offers a personalized risk calculator for patients with MPN. This risk score is sophisticated, using 63 clinical and genomic variables to predict overall risk of leukemic transformation, and event-free survival (Table 8) [20]. The previously mentioned scores, AIPSS-MF and AIPSSmol-MF, can also be used to assess leukemia-free survival [81,82].

### 4.4. Transplant Models

As mentioned earlier, transplant is the only potentially curative treatment for myelofibrosis at present. Several scoring systems have been developed to predict risk as transplant carries significant morbidity and mortality, including MIPSS70 and MIPSS70+ version 2 discussed previously [74,75]. However, a key limitation of these scores is that they focus primarily on disease biology and do not consider the patient’s overall health as well as the presence of comorbidities. Therefore, decisions regarding transplant, which are complex, must consider patient eligibility and transplant timing as well as disease-related factors.

The Myelofibrosis Transplant Scoring System (MTSS) score is used to predict survival post-transplant for patients with myelofibrosis (primary and secondary) based on older age (≥57 years), reduced performance status, thrombocytopenia, leukocytosis, human leukocyte antigen (HLA)-mismatched unrelated donor, *ASXL1* mutation, and a non-*CALR/MPL* genotype. Patients are then risk stratified into low, intermediate, high and very high risk [83]. Researchers further developed an improved and integrated MTSS score, highlighting the role of cytogenetics and molecular profile in predicting outcomes post-transplant (iMTSS). This was shown to be superior to existing tools including DIPSS, MIPSS70, MIPSS70+ version 2.0 and MTSS scores [84].

Due to genomics not being widely available at all centers, Tamari et al. developed a multivariate model, CIBMTR, using three independent variables to predict survival post-transplant. These variables included age > 50, hemoglobin level of <100g/L pre-allogenic stem cell transplant, and HLA-matched or -mismatched donor type, and were found to significantly influence survival [85]. Importantly, through incorporation of transplantation-related factors (donor availability), this score was superior in predicting transplant outcomes compared to the DIPSS and demonstrated that the donor source could overcome high-risk disease features [85]. Therefore, in current guidelines, it is recommended to combine DIPSS-Plus/MYSEC-PM together with MIPSS70+ version 2 and MTSS or CIBMTR to comprehensively assess patients under consideration for transplant [73].

A machine learning prognostic tool, the Myelofibrosis AlloRisk Stratifier, was recently developed by the European Society for Blood and Marrow Transplantation (EBMT) Chronic Malignancies Working Party to predict overall survival for myelofibrosis patients undergoing transplant. The variables used to calculate this risk included age, hemoglobin levels at the time of transplant, and donor type (these being considered critical independent predictors); comorbidities; and performance status as well as laboratory markers including blood blasts, leukocyte count, and platelet count [86]. Finally, the model accounted for the transplant protocol itself by factoring in conditioning intensity and the specific graft-versus-host disease (GVHD) prophylaxis used. This was also used to predict non-relapse mortality. The main advantage of this tool was that it took into consideration different aspects including patient factors and disease characteristics as well as transplant factors, and was able to robustly classify patients allowing for more personalized and effective patient management. Further research is required with this tool, with particular focus on examining the role of marrow fibrosis as well as variant allele frequency of driver mutations on transplant outcomes.

All risk scores discussed in this section are summarized in Table 12 alongside key variables, primary goal measured and their clinical utility.

## 5. Conclusions

Management of myeloproliferative neoplasms (MPNs) should be grounded in a comprehensive, risk-adapted framework rather than an exclusive focus on hematologic control. Contemporary prognostic models that integrate molecular data provide an additional layer of precision and, when combined with clinical expertise, enable more nuanced and individualized therapeutic decision-making within a shared decision-making paradigm. Importantly, molecular characterization now extends beyond mutational status to include allele burden, further refining risk stratification.

Looking ahead, emerging approaches incorporating machine learning and artificial intelligence hold promise in enhancing personalized risk prediction and dynamic disease modeling. These advances may facilitate more accurate patient selection for targeted interventions, with the overarching goals of improving patient-reported outcomes, minimizing thrombotic risk, delaying disease progression and leukemic transformation, and ultimately extending overall survival. Nevertheless, despite the promising performance of emerging molecular and artificial intelligence-based prognostic tools, prospective validation and real-world implementation studies are still required before these models can be routinely integrated into standard clinical practice.

## Figures and Tables

**Table 1:** Classic risk stratification in PV.

Risk Factor	Risk Group
Age < 60 years with no history of thrombosis or major hemorrhage	Low
Age ≥ 60 years and/or history of thrombosis or major hemorrhage	High

**Table 2:** Arterial thrombotic risk stratification in MPN (ARTS).

Risk Factor	Points	Relative Risk
Cardiovascular risk factors (hypertension, diabetes, smoking, or hypercholesterolemia)	3	3.00
Age ≥ 60 years at diagnosis	2	2.19
Prior arterial thrombosis at or before diagnosis	1	1.81
*TET2* or *DNMT3A* mutations	1	1.80

Low risk: 0–3 points with 0.37 arterial thrombosis risk (% patients/year); high risk: 4–7 points with 1.19 arterial thrombosis risk (%patients/year).

**Table 3:** Venous thrombotic risk stratification in MPN (VETS).

Risk Factor	Points	Relative Risk
Prior venous thrombosis at or before diagnosis	3	3.00
*JAK2*V617F variant allele frequency ≥ 50%	2	2.19

Low risk = 0 points, 5.8% (10-year probability of venous thrombosis), intermediate = 1 point, 9.5%, high risk = 2 points, 24.9%.

**Table 4:** Survival risk stratification in PV according to the MIPSS scale.

Risk Factor	Points
>60 years	2
Leukocytes > 11 × 10^9^/L	1
Abnormal karyotype	1
*SRSF2* mutation	2

Low (0–1 pts, median survival 25.3 years); intermediate-1 (2 pts, 18 years); intermediate-2 (3 pts, 10 years); high (≥4 pts, 5.4 years).

**Table 5:** Scoring of the IWG-MRT prognostic model in polycythemia vera.

Clinical Factor	Points
Age ≥ 67 years	5
Age 57–66 years	2
Leukocytes ≥ 15 × 10^9^/L	1
History of venous thrombosis	1

Low (0 points best survival); intermediate (1–2 points, intermediate survival); high (≥3 points, worst survival).

**Table 6:** AAA score.

Variable	Points
Age > 70 years	4
Age 50–70 years	2
Absolute neutrophil count ≥ 8 × 10^9^/L	1
Absolute lymphocyte count < 1.7 × 10^9^/L	1

Low (0–1 points, longer estimated survival ~47 y); intermediate-1 (2–3 points, intermediate estimated survival ~20.7 y); intermediate-2 (4 points, moderate estimated survival ~13.5 y); high (5–6 points, estimated survival ~8 y).

**Table 7:** Clinico-molecular prognostic model by Grinfeld et al. in polycythemia vera.

Domain	Variables Included in the Model
Demographic	Age at diagnosis
Clinical	History of thrombosis
Hematologic	Leukocyte count, platelet count
Organ-related	Splenomegaly
Genetic	Additional somatic mutations (e.g., *ASXL1*, *SRSF2*, *DNMT3A*, *TET2*)
Cytogenetic	High-risk cytogenetic abnormalities
Predicted outcomes	Overall survival; risk of progression to post-PV myelofibrosis or acute myeloid leukemia

**Table 8:** Summary of PV risk stratification scores.

Score	Predicts	Key Variables	Validated?	First Author	Year Published
Traditional (ELN)	Thrombosis	Age > 60, prior thrombosis	Yes		
ARTS	Arterial thrombosis	Age ≥ 60 years, prior arterial thrombosis, *TET2* or *DNMT3A* mutation	Yes	Hélène Pasquer	2024 [24]
VETS	Venous thrombosis	Prior venous thrombosis, *JAK2*V617F VAF ≥ 50%	Yes	Hélène Pasquer	2024 [24]
MIPSS-PV	Survival	Age, WBC, karyotype, *SRSF2* mutation	Yes	Ayalew Tefferi	2020 [27]
IWG-MRT	Survival	Age, WBC count, venous thrombosis history	Yes	Ayalew Tefferi	2013 [11]
Grinfeld (Genomic)	Personalized Outcome	63 variables (Genomic + Clinical)	Yes	Jacob Grinfeld	2018 [20]

**Table 9:** Summary of ET risk stratification scores.

Score	Predicts	Key Variables	Validated?	First Author	Year Published
Traditional (ELN)	Thrombosis	Age > 60, prior thrombosis/hemorrhage	Yes	Tiziano Barbui	2011 [35]
Revised IPSET-Thrombosis	Thrombosis	Age, prior clot, *JAK2* status, CV risk factors	Yes	Tiziano Barbui	2012 [36]
IPSET-Thrombosis	Thrombosis	Age, WBC count, prior thrombosis	Yes	Francesco Passamonti	2012 [37]
MIPSS-ET	Survival	Age, male sex, WBC, high-risk mutations (*SRSF2*, etc.)	Yes	Ayalew Tefferi	2020 [40]
ARTS/VETS	Arterial vs. Venous thrombosis	*JAK2* VAF, CV risk factors, male sex, leukocytosis	Yes	Hélène Pasquer	2024 [24]

**Table 10:** Prognostic factors and scoring systems (IPSS, DIPSS, DIPSS-Plus).

Prognostic Factor	IPSS (Points)	DIPSS (Points)	DIPSS-Plus (Points)
Age > 65 years	1	1	1
Constitutional symptoms	1	1	1
Hemoglobin < 10 g/dL	1	2	1
Leukocytes > 25 × 10^9^/L	1	1	1
Peripheral blood blasts ≥ 1%	1	1	1
Platelets < 100 × 10^9^/L	–	–	1
Transfusion dependence	–	–	1
Unfavorable karyotype: +8, −7/7q−, −5/5q−, i(17q), 12p−, inv(3), or 11q23 rearrangement	–	–	1

Prognostic model: risk group (points), median survival. IPSS (International Prognostic Scoring System): low risk (0 points), 11.3 years; intermediate-1 risk (1 point), 7.9 years; intermediate-2 risk (2 points), 4 years; high risk (≥3 points), 2.3 years. DIPSS (Dynamic IPSS): low risk (0 points), not reached; intermediate-1 risk (1–2 points), 14.2 years; intermediate-2 risk (3–4 points), 4 years; high risk (5–6 points), 1.5 years. DIPSS-Plus: low risk (0 points), 15.4 years; intermediate-1 risk (1 point), 6.5 years; intermediate-2 risk (2–3 points), 2.9 years; high risk (≥4 points), 1.3 years.

**Table 11:** Prognostic classification systems for survival in MF that include cytogenetic and molecular variables.

Prognostic Factor	MIPSS70 (Points)	MIPSS70+ v2 (Points)
Constitutional symptoms	1	2
Anemia < 10 g/dL (moderate–severe) ^a,b^	1	1–2
Leukocytes > 25 × 10^9^/L	2	
Platelets < 100 × 10^9^/L	2	
Circulating blasts ≥ 2%	1	1
Bone marrow fibrosis ≥ grade 2	1	
Absence of type 1 CALR mutation	1	2
HMR mutations ^c^	1	2
≥2 HMR mutations ^c^	2	3
Unfavorable karyotype ^d^		3
Very high-risk karyotype ^e^		4

The prognostic score is calculated from the sum of points of each model. ^a^ Moderate anemia: Hb 8–8.9 g/dL in women and 9–9.9 g/dL in men. ^b^ Severe anemia: Hb < 8 g/dL in women and <9 g/dL in men. ^c^ HMR, high-molecular-risk, mutations: In MIPSS70—include ASXL1, SRSF2, EZH2, IDH1/2; in MIPSS70+ v2—include the above plus U2AF1 Q157. ^d^ Unfavorable karyotype: Includes a single abnormality of very high risk (VHR), normal karyotype with one or two abnormalities such as del(20q), +8, chromosome 7 abnormality, 3q rearrangement, i(17q), or del(12p). ^e^ Very-high-risk karyotype: Abnormalities of chromosome 7, inv(3)/3q21, i(17q), 12p/12p11.2, or 11q/11q23, single or multiple autosomal trisomies other than +8. Prognostic model: median survival (points; years). MIPSS70: low risk (0–1), not reached; intermediate risk (1–4), 6.3 years; high risk (≥5), 3.1 years. MIPSS70+ v2: very low risk (0), not reached; low risk (1–2), 14.4 years; intermediate risk (3–4), 7.7 years; high risk (5–8), 4.1 years; very high risk (≥9), 1.8 years.

**Table 12:** Summary of risk stratification scores for myelofibrosis.

Category	Score	Primary Goal	Key Variables	Clinical Utility	Validation Status	First Author	Year Published
Clinical Factors	IPSS/ DIPSS	Survival Prediction	Age, Symptoms, Hb, WBC, Peripheral Blasts	Standard for routine monitoring; DIPSS can be used at any time.	Externally Validated (Standard of Care)	Francesco Passamonti	2010 [63,64]
Clinical Factors	DIPSS-Plus	Survival Prediction	DIPSS Variables + Platelets, Karyotype, Transfusion Dependency	Adds cytogenetic depth to the standard dynamic score.	Externally Validated	Naseema Gangat	2011 [67]
Molecular and Karyotype	MIPSS70+ version 2.0	Selection for Allo HSCT	HMR Mutations (*U2AF1*, *SRSF2*, *ASXL1*), Fibrosis Grade, Karyotype, Anemia	Identifies patients with high biological risk who need transplant.	Externally Validated (Multicenter)	Ayalew Tefferi	2018 [75]
Clinical and Molecular	MYSEC-PM	Survival in Secondary MF	Age, Hb, Blasts, Symptoms, Platelets, *CALR* Status	Validated specifically for post-PV and post-ET myelofibrosis.	Externally Validated (International)	Francesco Passamonti	2018 [77]
Machine Learning	AIPSS-MF/AIPSSmol-MF	Overall Survival & Leukemia Transformation	8 Clinical Variables (Age, Sex, etc.) ± Molecular Profiles	AI-driven; highly accurate for predicting transformation to AML.	Externally Validated (GEMFIN Registry)	Adrián Mosquera-Orgueira	2022 [81]
Transplant-Specific	MTSS	Post-HSCT Survival & Non-Relapse Mortality	Age, Performance status, *ASXL1*, Non-*CALR/MPL* genotype, HLA-match	The gold standard for molecularly integrated transplant timing.	Externally Validated	Nico Gagelmann	2019 [83]
Transplant-Specific	EBMT AlloRisk	Post-HSCT Overall Survival & Relapse	Age, Hb, Donor Type, Comorbidities, Conditioning, GVHD Prophylaxis	Machine learning tool that outperforms CIBMTR in OS prediction.	Externally Validated (5000+ Patients)	Juan Carlos Hernández-Boluda	2025 [86]
Transplant-Specific	CIBMTR	Post-HSCT Survival	Age > 50, Hb < 100 g/L, Donor Type (Matched vs. Mismatched)	A simplified, clinically focused model for rapid screening.	Externally Validated (Registry Data)	Roni Tamari	2023 [85]

## Data Availability

No new data were created or analyzed in this study. Data sharing is not applicable to this article.

## Abbreviations

The following abbreviations were used in this article:

AIPSS-MF	Artificial Intelligence Prognostic Scoring System for Myelofibrosis
Allo-HSCT	Allogeneic hematopoietic stem cell transplantation
ARTS	Arterial Thrombotic Score
DIPSS	Dynamic International Prognostic Scoring System
DIPSS-R	Revised Dynamic International Prognostic Scoring System
EBMT	European Society for Blood and Marrow Transplantation
ELN	European LeukemiaNet
ET	Essential thrombocythemia
GVHD	Graft-versus-host disease
Hb	Hemoglobin
HLA	Human leukocyte antigen
HMR	High-molecular-risk
iMTSS	Integrated Myelofibrosis Transplant Scoring System
IPSET-T	International Prognostic Score in Essential Thrombocythemia-Thrombosis
IPSS	International Prognostic Scoring System
IWG-MRT	International Working Group-Myeloproliferative Neoplasms Research and Treatment
MF	Myelofibrosis
MIPSS70/MIPSS70+ version 2	Mutation-Enhanced International Prognostic Score System for Transplantation Eligible-Aged Patients with Overt PMF
MIPSS-ET	Molecular International Prognostic Scoring System for Essential Thrombocythemia
MIPSS-PV	Mutation-Enhanced International Prognostic Systems for PV
MPN	Myeloproliferative neoplasm
MTSS	Myelofibrosis Transplant Scoring System
MYSEC-PM	Myelofibrosis Secondary to PV and ET-Prognostic Model
PMF	Primary myelofibrosis
PV	Polycythemia vera
RR6	Response to Ruxolitinib after 6 Months
SVT	Splanchnic vein thrombosis
TRM	Transplant-related mortality
VAF	Variant allele frequency
VETS	Venous thrombotic score
WBC	White blood cell
WHO	World Health Organization
